# Associations of Nutritional Health Risk With Cognitive Function in Older Korean Adults: The Mediating Roles of Lower Body Strength and Depression

**DOI:** 10.1111/ggi.70257

**Published:** 2025-11-18

**Authors:** Seamon Kang, Xiaoming Qiu, Hyunsik Kang

**Affiliations:** ^1^ College of Sport Science, Sungkyunkwan University Suwon Republic of Korea

**Keywords:** cognitive decline, depressive symptoms, nutritional risks, older adults, sarcopenia

## Abstract

**Aim:**

Malnutrition is associated with cognitive decline, but the causal relationship is unknown. This population‐based study of 9885 older Korean adults investigated the mediating roles of lower body strength (LBS) and depression on the relationship between nutritional health risk (NHR) and cognitive function.

**Methods:**

Cognitive function was evaluated using the Korean version of the Mini‐Mental State Examination. NHR was assessed using the Nutrition Screening Checklist. LBS was measured with a modified sit‐to‐stand test. Depression was defined as having a Korean version of the Geriatric Depression Scale (GDS‐K) score of ≥ 8. A mediation analysis was conducted to determine the relationship between NHR and cognitive function, with LBS and depression acting as mediators.

**Results:**

Age (*β* = −0.120, *p* < 0.001), education (*β* = 0.226, *p* < 0.001), smoking (*β* = −0.502, *p* < 0.001), income (*β* = 0.001, *p* < 0.001), marital status (*β* = −0.244, *p* < 0.001), NHR (*β* = −0.421, *p* < 0.001), LBS (*β* = −1.90, *p* < 0.001), and GDS‐K (*β* = −0.043, *p* = 0.0502) were significant predictors of cognitive function. NHR has an indirect effect on cognitive function through its impact on LBS (*β* = −0.013, 95% confidence interval [CI] = −0.020 to −0.006) and GDS‐K (*β* = −0.017, 95% CI = −0.033 to −0.007). NHR is also directly related to cognitive function (*β* = −0.067, 95% CI = −0.103 to −0.030).

**Conclusions:**

Our study findings suggest that NHR is associated with cognitive decline both directly and indirectly via its impact on LBS and GDS‐K.

## 1 Introduction

Cognitive decline, defined as the progressive loss of cognitive abilities in memory, attention, and processing speed, is associated with normal aging [1, 2]. Age‐related cognitive decline is an early indicator or precursor of neurologic disorders such as MCI and dementia [3]. Individuals, however, differ in their susceptibility to age‐related cognitive decline [4] due to the complex interplay of genetic, lifestyle, health, and environmental factors involved in its etiology [5, 6].

Malnutrition and sarcopenia are well‐established risk factors for cognitive decline [7, 8, 9]. Malnutrition can lead to cognitive decline in various ways, including decreased brain energy supply, impaired brain development and neuroplasticity, neuroinflammation, and neuronal cell death, especially in older adults [10, 11, 12]. Malnutrition in older adults can also lead to decreased physical function and increased mortality and morbidity [13, 14]. Sarcopenia, a condition characterized by loss of muscle mass and/or function, has been linked to cognitive decline in older adults through numerous mechanisms, including decreased myokine secretion, poor muscle function, oxidative stress, chronic inflammation, impaired blood vessel homeostasis, and additional pathways [15, 16, 17]. Sarcopenia significantly impacts lower body strength (LBS) and functional abilities, increasing the risk of falls and limiting mobility. LBS is crucial for mitigating these effects and maintaining independence in older adults. LBS has diagnostic value for the assessment and clinical management of sarcopenia [18]. Those previous findings suggest that malnutrition, sarcopenia, and cognitive decline are interconnected; malnutrition causes sarcopenia, which leads to cognitive decline in older adults [8, 19].

Depression is closely associated with cognitive decline in older adults [20]. These two conditions frequently coexist, and evidence suggests a bidirectional relationship [21]. On one hand, cognitive impairment may increase vulnerability to depressive symptoms due to functional decline and loss of independence. On the other hand, depression itself can contribute to cognitive deficits such as impaired memory, attention, and executive function, thereby accelerating cognitive decline. This reciprocal association highlights the complexity of determining the causal relationship between depression and cognitive impairment [22].

Overall, previous studies have linked cognitive decline to malnutrition, sarcopenia, and depression in older adults, but the causal relationship is still unclear [23, 24]. Understanding the complexities of this relationship is essential for developing effective intervention strategies to reduce cognitive decline that typically accompanies aging. In this study, we hypothesized that malnutrition impairs cognitive function both directly and indirectly via its impact on LBS and depression. We aimed to determine the mediating roles of LBS and depressive symptoms on the relationship between nutritional health risk (NHR) and cognitive function in older Korean adults.

## 2 Methods

### 2.1 Data Source and Study Participants

The Korea Longitudinal Study on Aging (KLoSA) was a national panel survey in South Korea from 2006 (the first wave) to 2020 (the eighth wave) to provide data for policy‐making and academic research. This cross‐sectional study used data from the 8th wave of the KLoSA participants aged 65 years and older (*n* = 10 097). Individuals with missing data on cognitive function, LBS, and GDS‐K (*n* = 212) were excluded. The remaining 9885 participants (3955 men/5930 women) were included in the final data analysis. KLoSA data were gathered through face‐to‐face interviews using Tablet‐PC Assisted Personal Interview. KLoSA data are accessible through the National Public Database (https://survey. keis. or. kr/eng/myinfo/login. jsp).

### 2.2 Data Collection

The measured variables of this study included cognitive function (outcome), NHR (exposure), LBS (mediator 1), and GDS‐K (mediator 2), as well as covariates.

#### 2.2.1 Mild Cognitive Impairment

Cognitive function was evaluated using a validated Korean version of the Mini‐Mental State Examination (K‐MMSE) optimized for screening dementia [25, 26]. The maximum score of K‐MMSE is 30 points. Individual cutoff values based on their age, sex, and education, as previously suggested [26], were adapted to assess mild cognitive impairment (MCI).

#### 2.2.2 Nutritional Health Risk, Lower Body Strength, and Depression

NHR was determined using the DETERMINE Your Nutritional Health Checklist [27]. The checklist includes 14 yes/no questions, and each question is worth between 1 and 4 points. This checklist covers eight key areas of NHR: disease, eating poorly, tooth loss/mouth pain, economic hardship, reduced social contact, multiple medicines, involuntary weight loss/gain, and needs assistance in self‐care. The total score, ranging from 0 to 21 points, was calculated by adding the values assigned to each question and categorized as good (0–2), moderate NHR (3–5), or high NHR (6 or more). The accuracy of the NHR screening tool has been validated in the older population [28].

LBS was assessed by conducting a sit‐to‐stand test [29], of which the performance assessment was modified for the KLoSA purpose. Briefly, participants were instructed to stand up quickly from a sitting position on a chair or bed with their arms folded across their chest. Performance was graded based on completeness (1 = completed, 2 = attempted but failed to complete, and 3 = no attempt).

Depressive symptoms were assessed using the Korean version of the 15‐item Geriatric Depression Scale (GDS‐K) [30]. Depression was defined as having a GDS‐K score of ≥ 8. Previous studies have tested and validated this cutoff score in psychiatric patients [31] and community‐dwelling older adults [30].

#### 2.2.3 Covariates

The covariates included age (years), sex (male vs. female), body mass index (kg/m^2^), educational background (elementary or less, middle/high school, or college/higher), annual income, marital status (never married, married and living with a partner, separated/divorced/widowed), smoking status (current/past smokers or nonsmokers), and chronic health conditions. The number of chronic conditions diagnosed by physicians was classified into three categories: none, one, and two or more.

### 2.3 Statistical Analysis

**FIGURE 1:**
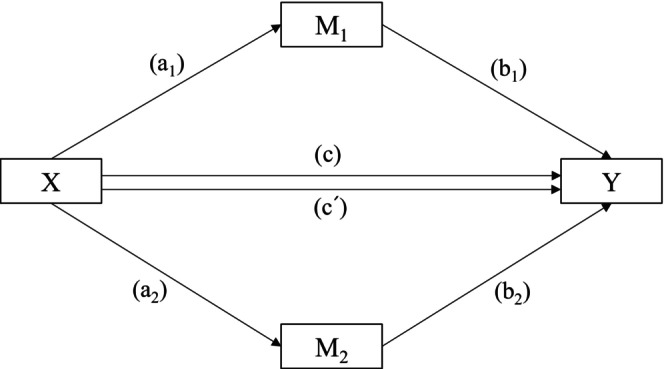
A mediation model with two mediators, where *X* is the independent variable, *M*_1_ and *M*_2_ are the two mediators, and *Y* is the dependent variable. There are two pathways, each one through only one mediator (H1: *X* → *M*_1_ → *Y* or H2: *X* → *M*_2_ → *Y*). c = c′ + a_1_b_1_ + a_2_b_2_; c = total effect of *X* on *Y*; c′ = direct effect of *X* on *Y*; a_1_b_1_ = indirect effect of X on Y through *M*_1_; a_2_b_2_ = indirect effect of *X* on *Y* through *M*_2_.

Quantile‐quantile plotting was used to determine the dataset's normal distribution. Student's *t*‐ and chi‐square tests were used to compare continuous and categorical variables, respectively. A multivariate linear regression with the enter method was conducted to determine significant predictors of cognitive function. The Hayes PROCESS Macro (Model 4) was used to conduct mediation analysis with two mediators. In this mediation regression analysis model, as illustrated in Figure 1, the independent variable (*X*) was NHR, the two mediators were *M*_1_ (LBS) and *M*_2_ (GDS‐K), and the dependent variable was *Y* (cognitive function). There are two pathways, each with only one mediator: H1 showing *X* causes *M*_1_, which in turn causes *Y* (*X* → *M*_1_ → *Y*), and H2 showing *X* causes *M*_2_, which then causes *Y* (*X* → *M*_2_ → *Y*). The statistical significance of the mediation model was tested using bias‐corrected 5000 bootstrapping and 95% confidence intervals (CIs). Statistical significance was tested using a nonzero value from the 95% bootstrapped CIs. All other statistical significance was determined at *p* = 0.05 using the statistical software PASW SPSS WIN 29.0 (SPSS Inc., Chicago, IL).

## 3 Results

Table 1 shows the descriptive statistics of the measured parameters according to cognitive function level. Participants with MCI were older (*p* < 0.001), more likely to be female (*p* = 0.012), less educated (*p* < 0.001), less likely to smoke (*p* < 0.001), and had a lower mean gross income (*p* = 0.014) than those with normal cognitive function. The distribution of education levels differed significantly between the MCI and normal cognition groups (*p* < 0.001), with the former having a higher proportion of individuals with middle or high school education but a lower proportion with college or higher education than the latter. Furthermore, individuals with MCI had a higher prevalence of multimorbidity (*p* < 0.001), depression (*p* < 0.001), increased NHR (*p* < 0.001), and poor LBS (*p* < 0.001) than those with normal cognitive function.

**TABLE 1:** Descriptive statistics of measured parameters by cognitive impairment status.

Variables	Total (*n* = 9885)	Normal (*n* = 6572)	MCI (*n* = 3313)	*p‐value*
MMSE‐K	24.3 ± 5.3	19.4 ± 5.5	26.8 ± 2.9	< 0.001
Age (years)	73.4 ± 6.5	73.0 ± 6.4	74.3 ± 6.5	< 0.001
BMI (kg/m^2^)	23.6 ± 2.6	23.6 ± 2.6	23.5 ± 2.7	0.092
Gender, *n* (%)				0.012
Men	3955 (40.0)	2572 (65.0)	1383 (35.9)	
Women	5930 (60.0)	4000 (67.5)	1930 (32.5)	
Education, *n* (%)				< 0.001
Elementary or lower	4413 (44.6)	3058 (46.5)	1355 (40.9)	
Middle or high school	4968 (50.3)	3125 (47.6)	1843 (55.6)	
College or higher	504 (5.1)	389 (5.9)	115 (3.5)	
Gross income (1000 won/year)	2696 ± 3966	2864 ± 4214	2364 ± 3396	< 0.001
Marital status, *n* (%)				0.233
Never married	41 (0.4)	29 (0.4)	12 (0.4)	
Married with a partner	5827 (58.9)	3910 (59.5)	1917 (57.9)	
Married without a partner	4017 (40.6)	2633 (40.1)	1384 (41.8)	
Smoking, *n* (%)	1079 (10.9)	750 (11.4)	329 (9.9)	0.014
Multi‐morbidity, *n* (%)	5293 (53.5)	3388 (51.6)	1905 (57.5)	< 0.001
Depression status, *n* (%)	4.7 ± 2.3	4.6 ± 2.1	4.9 ± 2.5	< 0.001
Not depressed	9009 (91.1)	6115 (93.0)	2894 (87.4)	
Depressed	876 (8.9)	457 (7.0)	419 (12.6)	
NHR, *n* (%)				< 0.001
Low NHR	7338 (74.2)	5045 (76.8)	2293 (69.2)	
Moderate NHR	1704 (17.2)	1045 (15.9)	659 (19.9)	
High NHR	843 (8.5)	482 (7.3)	361 (10.9)	
LBS, *n* (%)				< 0.001
Complete	7659 (77.5)	5439 (55.0)	2220 (22.5)	
Attempted but failed	1974 (20.0)	1024 (15.6)	950 (28.7)	
No attempted	252 (2.5)	109 (1.1)	143 (1.4)	

*Note:* Chronic disease was defined as the presence of a physician‐diagnosed disease. Depression was defined as having a score of 8 or higher on a Korean version of the 15‐item geriatric depression scale. Nutritional health risk (NHR) was assessed using the DETERMINE Your Nutritional Health Checklist. Lower body strength (LBS) was evaluated by sitting and standing on a chair five times consecutively.

Abbreviations: MCI, mild cognitive impairment; MMSE‐K, a Korean version of the Mini‐Mental Status Examination.

Table 2 shows the outcomes of a binary logistic regression analysis for NHR, LBS, and depression. People with moderate and high NHR had a significantly higher risk of having MCI (OR = 1.386, 95% CI = 1.240–1.550, and OR = 1.596, 95% CI = 1.376–1.850, respectively) than those with low NHR (Ref). Individuals who attempted but failed or did not attempt the five‐times‐sit‐to‐stand test were more likely to develop MCI (OR = 2.353, 95% CI = 1.956–2.830, and OR = 4.886, 95% CI = 4.022–5.934, respectively) than those who completed it (Ref). Similarly, depressed people had a higher risk of MCI (OR = 1.873, 95% CI = 1.626–2.158) than non‐depressed people (Ref).

**TABLE 2:** The odds ratios (ORs) and 95% confidence intervals (CIs) of mild cognitive impairment according to lower body strength, nutritional health risk, and depression.

		Model 1 OR [95% CI]	*p*	Model 2 OR [95% CI]	*p*
LBS	Complete	1		1	
	Attempted but failed	2.323 [1.939, 2.781]	< 0.001	2.353 [1.956, 2.830]	< 0.001
	No attempted	4.353 [3.632, 5.218]	< 0.001	4.886 [4.022, 5.934]	< 0.001
NHR	Good	1		1	
	At moderate risk	1.387 [1.244, 1.548]	< 0.001	1.386 [1.240, 1.550]	< 0.001
	At high risk	1.648 [1.425, 1.905]	< 0.001	1.596 [1.376, 1.850]	< 0.001
GDS‐K	Not depressed	1		1	
	Depressed	1.937 [1.685, 2.228]	< 0.001	1.873 [1.626, 2.158]	< 0.001

*Note:* Lower body strength (LBS) was evaluated by sitting and standing on a chair five times consecutively. Nutritional health risk (NHR) was assessed using the DETERMINE Your Nutritional Health Checklist. Depression was defined as having a score of 8 or higher on a Korean version of the 15‐item geriatric depression scale (GDS‐K). Model 1: no adjustment. Model 2: adjusted for all the measured covariates.

A multivariate linear regression with the enter method revealed that age (*β* = −0.120, *p* < 0.001), education (*β* = 0.226, *p* < 0.001), smoking (*β* = −0.502, *p* < 0.001), income (*β* = 0.001, *p* < 0.001), marital status (*β* = −0.244, *p* < 0.001), NHR (*β* = −0.421, *p* < 0.001), and LBS (*β* = −1.90, *p* < 0.001) were significant predictors of cognitive function, whereas GDS‐K (*β* = −0.043, *p* = 0.0502) showed only a non‐significant trend (Table S1).

**FIGURE 2:**
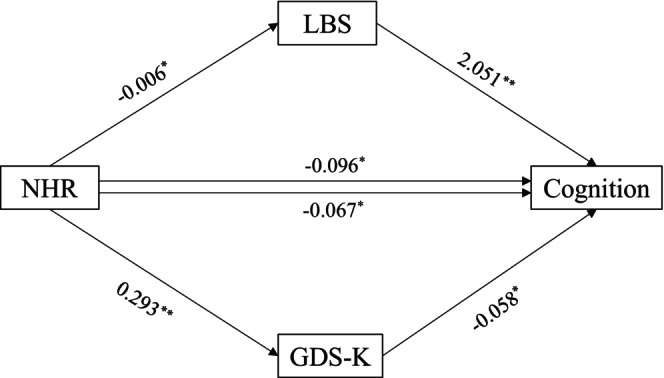
The relationship between nutritional health risk (NHR, independent variable) and cognitive function (dependent variable), with lower body strength (LBS) and the Korean version of the geriatric depression scale (GDS‐K) acting as two mediators. The asterisks indicate statistical significance: *p < 0.05 and **p < 0.01.

Figure 2 illustrates the outcomes of mediation analysis on the relationship between NHR and cognitive function, with LBS and GDS‐K acting as mediators. As a result, we found that although NHR is directly associated with cognitive function, the link between the two parameters is partially mediated by both LBS and GDS‐K. The calculated path coefficients indicate that about 70% of the total effect of NHR on cognitive function is attributable to a direct effect, while the remaining 30% is due to its effect on both LBS (13%) and GDS‐K (17%). As presented in Table 3, mediation analysis with 5000 bootstrapped samples revealed that NHR has an indirect effect on cognitive function through its impact on LBS (*β* = −0.013, SE = 0.004, 95% CI = −0.020 to −0.006) and GDS (*β* = −0.017, SE = 0.008, 95% CI = −0.033 to −0.007), supporting H_1_ and H_2_, respectively. Moreover, NHR is directly related to cognitive function (*β* = −0.067, SE = 0.019, 95% CI = −0.103 to −0.030) even when the two mediators are included in the regression model.

**TABLE 3:** Testing the mediating effects of lower body strength (LBS) and depressive symptoms on the relationship between nutritional health risk (NHR) and cognitive function.

Total effect (SE) [95% LLCI, ULCI]	Direct effect (SE) [95% LLCI, ULCI]	Paths	Indirect effect (SE) [95% LLCI, ULCI]	Conclusion
−0.096 (0.018) [−0.131, −0.062]	−0.066 (0.019) [−0.103, −0.030]	H1: NHR → LBS → COG	−0.013 (0.004) [−0.020, −0.006]	Partial mediation
		H2: NHR → GDS‐K → COG	−0.017 (0.008) [−0.033, −0.007]	Partial mediation

*Note:* The model was adjusted for age, sex, education, comorbidity, smoking, alcohol consumption, income, and marital status.

Abbreviations: CI, confidence interval; COG, cognitive function; GDS‐K, Korean version of geriatric depression scale; LBS, lower body strength; LLCI, lower limit CI; NHR, nutritional health risk; ULCI, upper limit CI.

## 4 Discussion

This population‐based cross‐sectional study of older Korean adults examined whether LBS and GDS‐K mediated the relationship between NHR and cognitive function. We found that NHR and LBS were significant predictors, while GDS‐K showed a non‐significant trend toward association with cognitive function in older Korean adults. Our findings from a mediation model regression analysis indicate that while NHR is directly related to cognitive decline, the relationship is also partially mediated by LBS and GDS‐K. Our findings suggest that in older adults, NHR, muscle weakness, and depressive symptoms are interrelated factors that contribute to cognitive impairment, highlighting the importance of concurrently addressing these conditions to support cognitive health.

Our findings support those of previous studies linking NHR, muscle weakness, and cognitive impairment in older adults. Malnutrition, poor eating habits, and dietary imbalances are prevalent in the older population. Older adults with nutritional risk are more likely to develop cognitive impairment and neurological disorders [32, 33]. Using data obtained from the Singapore Longitudinal Aging Studies, Lu et al. [34] evaluated the global nutritional risk in two cohorts of 3128 dementia‐free and 2640 cognitively normal Chinese older adults at baseline and after 3–5 years of follow‐up. They revealed that malnutrition was a significant predictor of cognitive decline and subsequent neurocognitive disorders such as MCI and dementia. By analyzing the data obtained from 806 Korean older adults aged ≥ 60 years who participated in the Korean Multi‐Rural Communities Cohort Study, which is a part of the Korean Genome Epidemiology Study, Kim et al. [35] found that a rice‐centered and unbalanced diet was a significant risk factor for cognitive impairment. In a cross‐sectional study of 806 community‐dwelling older Korean adults, Kim et al. [36] demonstrated that a high diet quality score was significantly and inversely related to the risk of MCI.

In addition to NHR, prior studies have shown that sarcopenia is an independent risk factor for cognitive decline in older adults [19, 37, 38]. Research in older Korean adults has also reported associations between sarcopenia and cognitive impairment [39, 40]. In the present study, we found that LBS and depressive symptoms partially mediated the relationship between NHR and cognitive function. These findings align with previous research indicating that the effect of nutritional status on cognitive impairment may be mediated by sarcopenia [8, 34]. Overall, both our study and prior evidence indicate that sarcopenia likely plays an important role in linking nutritional risk to cognitive decline, with poor nutritional status exacerbating the negative impact of sarcopenia on cognitive function [41].

Depressive symptoms are closely linked to poorer cognitive function in older adults and may play a mediating role in the relationship between NHR and cognition [42]. Previous studies have consistently shown that depression frequently coexists with cognitive decline and is associated with increased risk of neurological disorders such as MCI and AD [43, 44, 45, 46]. Moreover, depression often interacts with other health factors, including poor nutrition and sarcopenia, which can further exacerbate cognitive impairment [47]. Our findings extend this evidence by demonstrating that NHR is associated with lower cognitive function both directly and indirectly through depressive symptoms, highlighting the importance of addressing mental health in nutritional interventions to support cognitive outcomes in older adults.

This study has several limitations. First, the cross‐sectional design prevents causal interpretation of the mediating effects of LBS and GDS‐K on the relationship between NHR and cognitive function, since these variables may be bidirectionally interrelated. For example, cognitive decline can affect appetite, meal preparation, and physical activity, which in turn influence NHR and lower‐body strength. Prospective studies are needed to clarify these relationships. Second, both the GDS‐K and NHR rely on self‐reported information, which may be affected by cognitive impairment, language barriers, or reporting bias [48, 49]. Combining self‐report measures with structured assessments or objective nutritional evaluations would improve accuracy. Third, the accuracy of LBS measurements may vary depending on chair or bed height across participants, and this should be confirmed. Fourth, chronic health conditions such as obesity, hypertension, and diabetes may exacerbate the effects of malnutrition and sarcopenia, further increasing the risk of cognitive impairment. Future research should consider these comorbidities.

## 5 Conclusions

In conclusion, our findings indicate that older Korean adults with poor nutritional status are more likely to suffer from muscle weakness and depression, which can contribute to cognitive decline. These results suggest that targeting even a single factor might yield broader benefits, as these risk factors are interconnected.

## Author Contributions

S.K., X.Q., and H.K. conceptualized the study. S.K., X.Q., and H.K. organized the data collection process. S.K., X.Q., and H.K. conducted the data analysis and drafted the methodology section. All the authors wrote the conclusion section together and contributed to the revision of the final version of the article.

## Ethics Statement

The Institutional Review Board of the Korea Institute for Health and Social Affairs reviewed and approved the survey (approval no. 2020‐36). Informed consent was obtained from all participants. The survey was conducted under the Declaration of Helsinki.

## Conflicts of Interest

The authors declare no conflicts of interest.

## Supporting information


**Table S1:** Linear regression for cognitive function.

## Data Availability

All data used in this study are available upon reasonable request from the corresponding author.
