# Supplementary material for: Associations of Nutritional Health Risk With Cognitive Function in Older Korean Adults: The Mediating Roles of Lower Body Strength and Depression
Source: Geriatr Gerontol Int. 2025 Nov 18;25(12):1846–52. doi: 10.1111/ggi.70257 (PMC12719135; doi:10.1111/ggi.70257)
Supplement: Supplementary file 1 — Table S1: Linear regression for cognitive function. [file GGI-25-1846-s001.docx]

| Supplemental Table 1. Linear regression for cognitive function. | | | | | | |
| --- | --- | --- | --- | --- | --- | --- |
| Variables | Unstandardized β (SE) | t | 95% CI | | VIF | *p*-value |
|  |  |  | LLCI | ULCI |  |  |
| Age | -0.120 | -12.859 | -0.139 | -0.102 | 1.597 | <0.001 |
| Sex | -0.028 | -0.242 | -0.255 | 0.199 | 1.387 | 0.809 |
| Education | 0.226 | 15.154 | 0.197 | 0.256 | 1.583 | <0.001 |
| Marriage | -0.244 | -2.783 | -0.416 | -0.072 | 1.169 | <0.001 |
| Income | <0.001 | 5.934 | <0.001 | <0.001 | 1.095 | <0.001 |
| Smoking | -0.502 | -3.028 | -0.828 | -0.177 | 1.149 | 0.002 |
| # of chronic diseases | 0.019 | 0.525 | -0.052 | 0.090 | 1.192 | 0.600 |
| NHR | -0.421 | -5.030 | -0.586 | -0.257 | 1.943 | <0.001 |
| LBS | -1.900 | -13.871 | -2.168 | -1.631 | 1.848 | <0.001 |
| GDS-K | -0.044 | -1.946 | -0.087 | 0.001 | 1.069 | 0.0502 |
| NHR, nutritional health risk; LBS, lower body strength; GDS, Korean version of geriatric depression scale. | | | | | | |

**Supplement materials**
